# Promoting health equity through linguistic justice: mitigating embedded bias of large language models

**DOI:** 10.1038/s41746-026-03093-4

**Published:** 2026-08-08

**Authors:** Weipeng Han, Sol Richardson, Jiming Zhu, Yuanli Liu

**Affiliations:** 1 School of Health Policy and Management, Chinese Academy of Medical Sciences & Peking Union Medical College, Beijing, China; 2 Vanke School of Public Health, Tsinghua University, Beijing, China; 3 China Medical Board, Beijing Office, Dongcheng District, Beijing, China

**Keywords:** Health care, Social sciences

## Abstract

The application of Large Language Models (LLMs) in healthcare prompts critical reflection on the impact of linguistic justice on health equity. Disparities in data availability across languages during the training and evaluation phases of LLMs result in significant performance variations. These disparities risk reinforcing existing inequities in healthcare access and outcomes. This study outlines strategies for integrating linguistic justice into LLM assessment frameworks, advancing health equity and global health.

## Large language models and health equity

Large Language Models (LLMs) have the potential to revolutionize the global health landscape, enhance healthcare practitioner expertise, and foster a more efficient, accessible, and patient-centered healthcare system^1^. While LLMs such as ChatGPT still require expert oversight to refine outputs, their demonstrated capacity to deliver accurate, actionable medical information positions them as transformative tools for augmenting clinical workflows and patient education^2^. Linguistic justice acknowledges language as a sociostructural determinant of opportunities and experiences, advocating for equitable access to linguistic capital across linguistic divides^3^. The growing application of LLMs in healthcare settings prompts us to recognize the emerging nexus between linguistic justice and global health.

Multilingualism, rather than monolingualism in English or dominant national languages, better achieves the core objectives of linguistic justice^3^. Despite the potential of LLMs’ extensive multilingual resources to significantly reshape healthcare accessibility and outcomes, linguistic and cultural biases embedded in LLMs risk exacerbating health inequities, particularly for marginalized populations^4^.

## Languages affect the performance of LLMs?

There are also significant disparities in the availability of natural language text across different linguistic groups within existing natural language corpora. This poses a significant challenge given their vital role as resources for LLMs training. The Responsible Open-science Open-collaboration Text Sources (ROOTS) corpus, a large-scale high-quality text dataset, comprises 46 natural languages. Of note, English (30.03%), Simplified Chinese (16.16%), French (12.9%), and Spanish (10.85%) collectively constitute 68% of the entire corpus^5^. The Multilingual Medical Corpus (MMedC) is a large-scale, domain-specific dataset comprising 25.5 billion tokens across six major languages: English (41.4%), Chinese (18.6%), French (17.1%), Spanish (8.0%), Russian (7.5%), and Japanese (6.6%)^6^. The glaring disparity in data availability inevitably leads to discrepancies in the foundational capabilities of LLMs across different linguistic contexts, raising grave concerns over linguistic inequities that may propagate through their downstream applications. Due to the limited availability of training data for low-resource languages, LLMs often exhibit suboptimal performance when processing non-Latin scripts^7^.

**Fig. 1:**
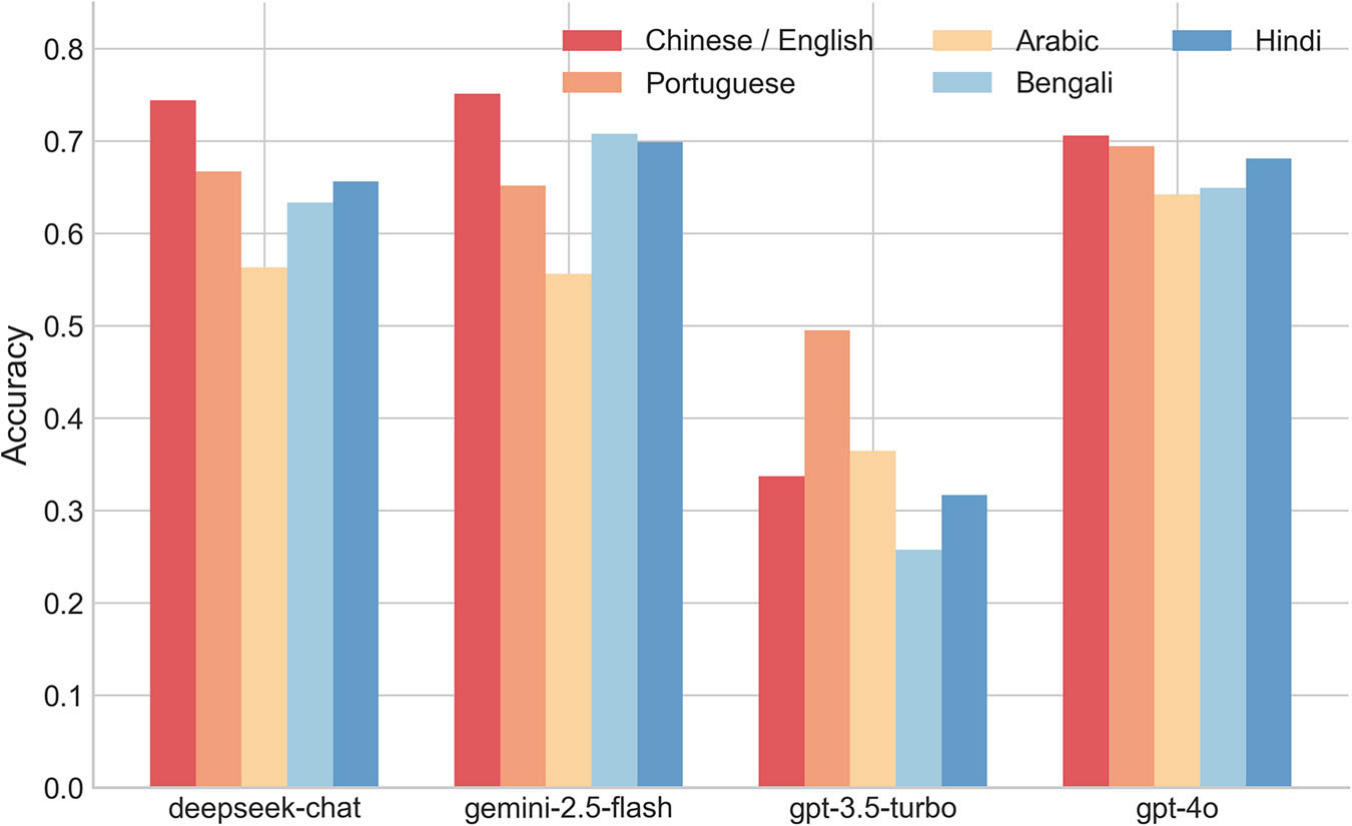
Accuracy of LLMs in Different Languages for Medical Examination Tasks.

LLMs exhibit notable accuracy declines when performing medical licensing exams in low-resource languages. LLMs such as GPT-4o demonstrate exceptional translation capabilities, including robust performance in certain low-resource languages^8^. This has led to the belief that leveraging the powerful translation abilities of LLMs can fully compensate for the scarcity of training data in specific languages. To test this hypothesis, we designed an experiment consisting of three stages: random question selection, question translation, and LLM-based answering. First, we randomly sampled 10% of the Simplified Chinese and English questions from the MedQA test set, yielding a total of 442 questions from medical licensing exams. Second, we translated these questions into four languages (Arabic, Bengali, Hindi, and Portuguese) using GPT-4o. These four languages were selected as representative low-resource languages, as they are among the ten most widely spoken languages worldwide, yet have only limited data available in the ROOTS and MMedC datasets. In the ROOTS corpus, English and Chinese data are several times larger than Hindi (24.6B bytes, 5.1% of English; 9.4% of Chinese), Bengali (18.6B bytes, 3.8% of English; 7.1% of Chinese), Arabic (74.9B bytes, 15.4% of English; 28.7% of Chinese), and Portuguese (79.3B bytes, 16.3% of English; 30.4% of Chinese)^5^. Moreover, the MMedC dataset does not contain any Hindi, Arabic, Bengali, or Portuguese data. Third, we evaluated four different LLMs by tasking them with answering physician licensing exam questions in each of the translated languages. The results are presented in Fig. 1. Using the Chinese and English question performance as reference baselines, we observed varying degrees of accuracy decline when LLMs answered questions in low-resource languages. For Portuguese, the accuracy decreased by only 0.73% relative to the baseline. However, for Arabic, accuracy declined by 10.29%; for Bengali, by 7.24%; and for Hindi, by 4.64%. The relatively small decline for Portuguese reflects its close lexical and morphological ties to high-resource Romance languages, which enable effective cross-linguistic transfer despite limited direct data^9^. By contrast, the sharper declines for Arabic, Bengali, and Hindi highlight how greater typological distance and underrepresentation in training corpora exacerbate disparities in LLMs’ performance. These findings empirically indicate that individuals interacting with LLMs in low-resource languages may face elevated health risks due to reduced reliability in medical advice. A limitation of our tests is that translation errors in LLMs may exacerbate performance disparities across languages.

Performance evaluation of four LLMs on 442 multilingual physician licensing questions sampled from MedQA and translated into four languages (Hindi, Arabic, Bengali, Portuguese) using GPT-4o, with Chinese/English serving as baselines. The bar chart displays mean accuracy scores (y-axis) across the four LLMs (x-axis: deepseek-chat, gemini-2.5-flash, gpt-3.5-turbo, and gpt-4o). Distinct colors represent different language groups.

## Linguistic justice initiative for AI in healthcare

Practitioners applying LLMs have encountered barriers in low-resource languages, highlighting the importance of linguistic justice in promoting health equity to LLM-driven services. For example, MkulimaGPT, a sensor‑based maize disease detection LLM delivered via mobile messaging, diagnoses issues from uploaded photos and guides farmers’ interventions^10^. To address the scarcity of Swahili training data, the development team secured funding from a private charitable foundation to compile high‑quality, locally sourced linguistic resources. While such measures have shown promise in specific contexts, the challenges are even more acute in healthcare, where linguistic justice can directly affect diagnostic accuracy, patient safety, and public health outcomes.

The integration of LLMs into healthcare decision-making reveals a critical dilemma in balancing linguistic accessibility with geographic specificity of medical knowledge. Healthcare decision-making inherently relies on localized clinical guidelines and geographically bounded epidemiological patterns. If LLMs were to strictly adhere to clinical practice guidelines for a particular country when responding to user queries, this could perpetuate a form of bias. Such a bias would undermine efforts toward achieving equity in global health. Therefore, responses of LLMs should therefore be tailored to align with the specific clinical practice guidelines prevalent in the user’s location. Providing appropriate prompts or establishing a service based on retrieval-augmented generation technology using localized clinical guidelines could serve as a potential solution. At the core of this challenge lies the imperative to ensure that LLMs are trained on sufficiently diverse collections of clinical guidelines from multiple countries and regions, with proportional representation in supervised fine-tuning datasets, and to guarantee that evaluation frameworks explicitly account for such variability to avoid systematic inequities.

We argue that the global health community must integrate linguistic justice in evaluations of LLMs, incorporating standardized clinical data ontologies and terminology harmonization to ensure AI-assisted healthcare adapts to regional practice guidelines while mitigating biases from semantic variance. Although existing studies have attempted to assess racial and ethnic biases, there remains a paucity of research addressing linguistic justice^11^. This gap highlights the current neglect of linguistic justice in health technology assessment and policy guidance. Therefore, we should strengthen governance to ensure LLMs uphold linguistic justice with transparency and accountability. First, a multidisciplinary expert review committee should be convened, comprising clinical experts, artificial intelligence (AI) researchers, ethicists, health policymakers, and representatives from diverse geographical regions. Second, a comprehensive database encompassing clinical practice guidelines from various regions should be created and regularly updated, probably with commercial licensing mechanisms to facilitate its utilization and sustainability. Third, it is essential to develop an expandable benchmark for evaluating the multilingual medical capabilities of LLMs. This entails constructing a large-scale, multilingual dataset annotated by clinical experts, accompanied by a corresponding framework that supports fine-grained error analysis^12,13^. Moreover, such datasets and analytical tools can be designed to reflect regional clinical guidelines. Fourth, systems should be implemented to gather feedback from healthcare professionals and patients on the relevance and effectiveness of model responses. Fifth, evaluation results and the measures taken to address issues identified should be published periodically. Sixth, exploration of modeling techniques is warranted, including cross-lingual transfer learning, and the inclusion of multiple languages in reinforcement learning from human feedback pipelines for mitigating language bias during model training and evaluation^14,15^.

To achieve the mission of developing Artificial General Intelligence (AGI) for the benefit of all humanity, it is imperative that stakeholders worldwide promptly establish a global technological innovation cooperation framework for health equity. A report from the United Nations reveals that 100 firms, predominantly headquartered in the United States and China, account for approximately 40% of global private investment in AI research and development^10^. Without a cooperative framework for inclusive data-sharing and equitable resource allocation, AGI advancements could exacerbate global health disparities. However, in recent years, we have witnessed a concerning trend of technology decoupling, severely impeding global technological innovation and collaboration. Ongoing technology decoupling, including in areas ranging from semiconductor supply chains to algorithmic transparency, threatens to create barriers for developing countries seeking to develop or adopt AI-assisted healthcare solutions.

In conclusion, AGI development—left unguided by a cooperative framework—threatens to replicate and amplify global health inequities. At the current stage of AI development, there is an opportunity to promote health equity through several critical initiatives. By institutionalizing inclusive governance, resource-sharing, and risk management, stakeholders can combine AGI toward its transformative potential as health equity. An equally important dimension of health equity involves linguistic and cultural inclusion, particularly in capturing knowledge from traditional, complementary, and alternative medicine. Incorporating these heritages from different nations into LLMs would help enrich the existing body of knowledge about the science and art of healing around the world as well as make healthcare practice more culturally sensitive. Although LLM-assisted healthcare is new, the underlying challenges in global health cooperation are as old as history. The establishment of the World Health Organization in the post-war era, or the global eradication of smallpox through international cooperation demonstrate that cross-border collaboration on health, and the mission to ensure global equity, are both achievable and imperative. Even in the 11th century, medical scholars such as Constantine the African systematically translated Arabic medical literature into Latin, bringing about a shift in medical practices in medieval Europe^16^. In the 21st century, physicians, scholars, and engineers should likewise commit to taking action on behalf of the more than five billion individuals worldwide who speak low‑resource languages.

## Data Availability

The datasets generated and/or analyzed during the current study are available in the Figshare repository, 10.6084/m9.figshare.29938535.
